# Ensuring Fact-Based Metabolite Identification in Liquid Chromatography–Mass Spectrometry-Based Metabolomics

**DOI:** 10.1021/acs.analchem.2c05192

**Published:** 2023-02-15

**Authors:** Georgios Theodoridis, Helen Gika, Daniel Raftery, Royston Goodacre, Robert S. Plumb, Ian D. Wilson

**Affiliations:** † Department of Chemistry, Aristotle University of Thessaloniki, Thessaloniki 54124, Greece; ‡ Biomic AUTh, Center for Interdisciplinary Research and Innovation (CIRI-AUTH), Balkan Center B1.4, 10th km Thessaloniki-Thermi Rd., P.O. Box 8318, Thessaloniki 57001Greece; § FoodOmicsGR, AUTh node, Center for Interdisciplinary Research and Innovation (CIRI-AUTH), Balkan Center B1.4, 10th km Thessaloniki-Thermi Rd, P.O. Box 8318, Thessaloniki 57001, Greece; ∥ Laboratory of Forensic Medicine and Toxicology, Department of Medicine, Aristotle University, Thessaloniki 54124, Greece; ⊥ Northwest Metabolomics Research Center, 850 Republican St., Seattle, Washington 98109, United States; # Mitochondria Metabolism Center, Anesthesiology and Pain Medicine, University of Washington, Seattle, Washington 98109, United States; ∇ Centre for Metabolomics Research, Department of Biochemistry and Systems Biology, Institute of Systems, Molecular and Integrative Biology, University of Liverpool, BioSciences Building, Crown St., Liverpool, L69 7ZB, United Kingdom; ○ Scientific Operations, IMMERSE, Waters Corporation, Cambridge 02142, Massachusetts United States; ◆ Division of Systems Medicine, Department of Metabolism, Digestion and Reproduction, Imperial College London, Hammersmith Campus, London W12 0NN, United Kingdom

## Abstract

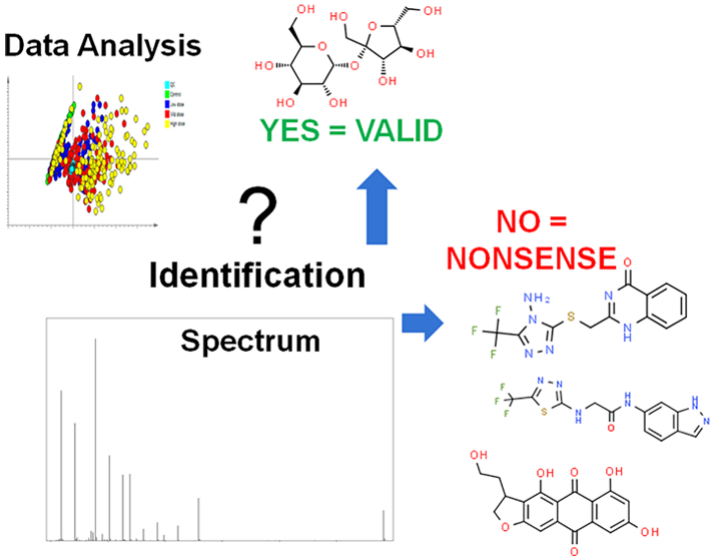

Metabolite identification represents a major bottleneck in contemporary metabolomics research and a step where critical errors may occur and pass unnoticed. This is especially the case for studies employing liquid chromatography–mass spectrometry technology, where there is increased concern on the validity of the proposed identities. In the present perspective article, we describe the issue and categorize the errors into two types: identities that show poor biological plausibility and identities that do not comply with chromatographic data and thus to physicochemical properties (usually hydrophobicity/hydrophilicity) of the proposed molecule. We discuss the problem, present characteristic examples, and propose measures to improve the situation.

## Introduction

**Figure 1:**
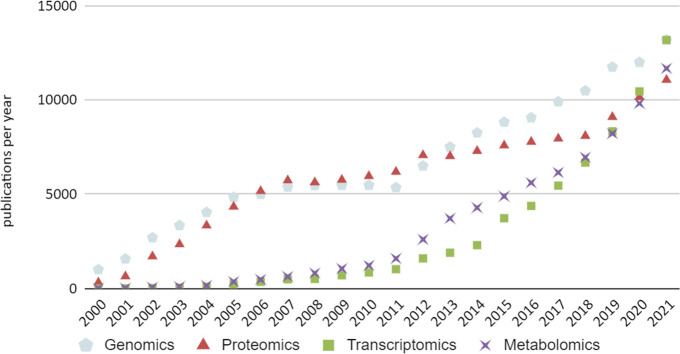
Plot of the number of publications per year for the four major omics disciplines. A significant increase is observed for transcriptomics and metabolomics publication in the last 5 years so both fields have caught up with genomics and proteomics which 15 years ago represented a several-fold larger number of publications. Search was made in Scopus (August 2022).

Metabolic phenotyping/profiling (metabolomics/metabonomics) is the broad study of metabolites and metabolism within biological systems and is now considered an emergent science. Within this area, publications in liquid chromatography–mass spectrometry (LC-MS) continue to increase, and the methodology is now routinely used in a wide range of scientific fields, including applications to environmental, food, and nutrition sciences, biomedicine, clinical investigations, and epidemiology, as well as plant and microbial sciences.^1−3^ Metabolomics continues to gain new adherents in other disciplines and has expanded with (seemingly) a faster annual growth rate in comparison to other established omics fields, thus closing the gap in publication numbers (see Figure 1 for a plot of number of “omics” publications per year since 2000).

This expansion is associated with an increased number of researchers becoming active in the field and a concomitant increase in conferences and publications in scientific journals. This growth has resulted in a large influx of new scientists to this field, with varying backgrounds, including those with perhaps limited experience in analytical science, data processing, and statistics, with many researchers performing metabolomics for the first time. As a result, it is inevitable that a significant proportion of these new researchers will have (in their initial studies) little experience on the conduct of metabolomic studies and an imperfect understanding of certain fundamental technical aspects that are key to obtaining reliable results in this demanding interdisciplinary field. Although the Metabolomics Standards Initiative (MSI) published a series of minimum reporting standards across the whole metabolomics workflow in the journal *Metabolomics* in 2007,^4,5^ many new researchers are not familiar with the requirements for reproducible analytical processes involved in the experiments and, in particular, those around robust and accurate metabolite identification.^5^

In addition, the expanded application of metabolomics is resulting in papers being submitted to journals with more general scientific content and emphasis. Indeed, broad-based journals such as *PLOS One* or *Scientific Reports* are currently among the top destinations for the publication of metabolomics research. Furthermore, topical “dedicated” journals specialized in particular fields, e.g., clinical journals, also publish articles focused on metabolomic biomarker research. Such submissions are usually handled by editors and reviewers with research viewpoints specifically aligned to the journal’s central topic that can range from clinical sciences to water and environment research. Thus, the scrutiny of technologies and methods employed in the investigation described in the manuscript may not be at the same level of that provided by specialized, field-specific, metabolomics-oriented journals.

Another increasing trend is outsourcing LC-MS analysis to a third party, core facility, or some other private enterprise, which then provides reports with finalized results. In some cases, little information is provided on methods/spectral libraries (which are often “proprietary”), as well as on quality control and the confirmation of the findings, etc. Unfortunately, raw data are often kept with the third party and may not reach the authors of the papers and so are not deposited in metabolomics repositories such as MetaboLights or Metabolomics Workbench. This approach does not comply with the minimum standards of reporting scientific research or the principles of FAIR (Findable, Accessible, Interoperable, and Reusable).^6,7^

From our years of experience as practitioners within analytical chemistry, scientific journal editors, and reviewers dealing specifically with MS-based metabolomics, we have handled a large number of metabolomics research manuscripts. Examination of these papers has revealed that problems with submitted work may occur at various stages beginning with study design, preanalytical steps, LC-MS analysis, post-analytical data pretreatment and statistics, metabolite identification, pathway analysis, and indeed the biochemical, mechanistic, and translational aspects of a study. These problems often result in important shortcomings which, in some cases, are identified in the review process of a manuscript, often leading to the outright rejection of the work. Unfortunately, it is clear from the literature that in many other cases flawed work passes unnoticed and is published despite results that are obviously dubious to those experienced in the field. The publication of such works is a serious problem because it can lead to much wasted effort and resources as other researchers, who are similarly not appropriately versed in the metabolomics workflow, may attempt to use these findings or validate them. In consequence, badly conducted studies can result in a loss of confidence in the value of metabolic phenotyping as a method for discovering biomarkers, identifying key metabolic perturbations, and understanding important biological phenomena. Moreover, such studies are wasteful of resources and thus not (environmentally) sustainable.

We also know from numerous discussions with colleagues that these observations are the common experience of many of our fellow researchers. Over the past several years, there have been several initiatives aimed at improving standards in metabolomics and lipidomics such as the Metabolomic Standards Initiative,^5^ while other researchers have published similar considerations independently.^8,9^ Recently, the need for improved approaches and standards led to the formation of the Metabolomics Quality Assurance and Quality Control Consortium (mQACC)^10−12^ and the Lipidomics Standards Initiative.^13,14^

Perhaps the most alarming recurring issue in our view is the erroneous and outright implausible identification of metabolic features which are proposed as potential biomarkers. Above and beyond false discoveries, these errors can and should be eliminated by improved methods that can be readily implemented by the field. In this commentary, we provide some examples of common errors in metabolite identification found in manuscripts both rejected and published. Our aim is not to name and shame but rather to increase awareness of authors and particularly reviewers and editors who represent the last lines of defense against spurious/misleading results and thereby reduce the incidence of incorrectly annotated or “identified” metabolites in the literature. We have also made some recommendations as to improvements in the workflow that should help to reduce such errors.

## The Problem

If mechanistic biochemical insight is to be extracted from LC-MS data obtained in untargeted metabolic phenotyping, then it is essential that the proposed potential biomarkers are fully identified. Only with confident identification can hypotheses be constructed and models be developed. It is only after reaching unequivocal Level 1 identification (as proposed by MSI (Metabolomics Standards Initiative and other researchers^5,15^), based on matching two orthogonal analytical characteristics (e.g., accurate mass, NMR shift, retention time (*t*_R_), fragmentation pattern in MS^*n*^) to a standard analyzed on the same instrument as the study reported, that metabolomic findings can be compared across laboratories and validated in subsequent studies and thus potentially be translated into applications in, e.g., clinical science.

However, as we indicated above, the generation of LC-MS data and metabolite identification is now not always performed by scientists with expertise in analytical separations and MS. In addition, as metabolomics research enters diverse fields, various types of specimens are analyzed, e.g., cell cultures, foods, insects and their parts, tissues from plants, and “unusual” biological fluids and tissues. These novel metabolomes are, as yet, not fully described and catalogued. It can be the case that researchers match their metabolomics data against species that are not the subject of their area or are not well represented in current databases, a fact that adds further issues in terms of provenance of the “identified” metabolite. As a result, researchers may find themselves having to analyze and interpret complex LC-MS data sets to detect and identify potential metabolic biomarkers when their expertise and knowledge does not equip them for the task. This is by no means an ideal situation for the reliable discovery and identification of unknown analytes.

Another alarming observation, as revealed by a recent meta-analysis of LC/MS-based metabolomic studies,^16^ is that only a small proportion of these works (ca. 20%) had employed reference standards to verify proposed metabolite identifications to MSI Level 1. Indeed, this meta-analysis showed that, in the majority of the submitted papers, identification and subsequent reporting of such “annotations” were mostly (if not entirely) based on the similarity of the detected mass-to-charge (*m*/*z*) values reported in public databases. This strategy is dangerously simplistic, as in such cases metabolite identification is often based on a single value, namely, the detected mass of the feature. In the worst cases, the comparisons can be made not to measured values of actual compounds but to the predicted/calculated mass of an analyte. Even when ultrahigh resolution MS instruments have been used, biology is replete with metabolites that are either isomers, enantiomers, or isobaric molecules. To have an indication of the numbers, a search in Chemspider for a small molecule with a monoisotopic mass of 118.0003 amu with a mass defect of 0.001 amu (that is, for molecules that range from MM ≥ 117.9993 to MM ≤ 118.0013) will provide 14 hits. Additional MS information, including fragmentation data, are therefore essential for confident identification, but frequently reference standard data are often not available to provide this. Indeed, it is important to be aware that while some databases do contain thousands of experimental MS and MS/MS spectra, obtained from reference standards (e.g., METLIN Gen2 reports the use of 860,000 such reference standards), for other databases (e.g., the HMDB) reference standard-derived spectra make up a relatively small proportion of the total, and the majority of the input data are from predicted spectra (as is clearly indicated on the HMDB website: https://hmdb.ca/statistics).

An additional complication is that the analysis of complex samples often results in peak coelution adding interfering MS peaks to the spectra of metabolites of interest. These “chimeric” spectra complicate spectral matching to library spectra. Apart from sample reanalysis using an LC system with greater resolving power or the use of columns with another selectivity, various approaches during data acquisition, above and beyond MS/MS etc., can be used to attempt overcome this problem. These center on various data independent acquisition (DIA) methods reviewed in Wang et al.,^17^ including SWATH-MS (sequential window acquisition of all theoretical mass spectra)^18^ and SONAR^19^ which rely on standardized data acquisition with retrospective mining of the data to extract spectra. One promising and rapidly emerging separation-based approach to improving MS data quality in metabolomics is to employ ion mobility (IM) enabled instrumentation to provide an orthogonal separation (based on the shape of the ionized molecules) to resolve coeluting species. An additional benefit is that IM can also provide a measurement of an analyte’s collision cross section (CCS).^20^ These CCS values then provide useful information supporting metabolite characterization through rapidly expanding databases (of both measured and calculated values) such as the ion mobility collision cross-section atlas for known and unknown metabolite annotation in untargeted metabolomics.^21^

In addition, in untargeted analysis, it is critical that researchers verify that the mass for the feature under investigation does indeed correspond to the molecular mass of the analyte and not the mass of an adduct, or results from the fragmentation of a larger molecule in the ion source. Unfortunately, this level of care is frequently not evident in manuscripts submitted for review, or indeed in many publications. In some cases, even the retention time (*t*_R_) for the LC is ignored, which is a significant oversight as these data provide valuable (orthogonal) information on the hydrophobicity/hydrophilicity of the metabolite being measured (see below).

Such practices are clearly unacceptable and is certainly not in line with existing guidelines such as those provided by, e.g., the Metabolomics Society and others, as well as guidelines from regulatory authorities such as the U.S. Food and Drug Administration (FDA) and European Medicines Agency (EMEA). See, for example, the FDA guidance on bioanalysis for industry,^22^ and more specifically EC decision 657/2002. The EC decision, which is used as a standard for food analysis, specifies that identification should be based on at least two orthogonal measurements, such as (retention time) *t*_R_ and a mass spectrum. The steps needed to accept or reject the identification of an analyte are explicitly described, and numerical evaluation is proposed. Three or four identification points are needed (e.g., *t*_R_, precursor and one product ion) to agree in the comparison of data obtained from the analysis of a real sample versus a fortified sample and a reference standard. Analyses are done on the same instrument and are “read” typically by highly experienced analytical scientists using a single software platform. This is as close to an ideal scenario in comparison to the approach currently prevalent in metabolic phenotyping research, where often comparisons are made in different laboratories, samples, instruments, software, and analytical conditions, which renders results hardly comparable.

Unfortunately, although these facts and policies have been known for some time, there is no enforcement of a similar level of scrutiny for metabolite identification in metabolomics analysis, and in contrast, we often observe poor analyte identification. Many papers found in the current literature exhibit what appears to be complete disregard of this important experience and guidance, and indeed, the majority of “identifications” are based largely on only an uncritical acceptance of comparisons of full scan LC-MS data with the MS data available from public databases. While there are many problems with such an approach, several major potential sources of problems are as follows: (1) The experimental MS data are collected under very different experimental conditions compared to those in the database. (2) As discussed earlier, a very large proportion of the data in some of the public libraries are not experimental but are largely based on predictions made using *in silico* tools, and several metabolomics data treatment software platforms are set to search in these databases as their default setting. (3) Oftentimes metabolites from a species of interest are putatively identified using databases that contain metabolites from another species, many species, or even the entire known chemical space. (4) The analysis of metabolites from human biosamples is complicated by the fact that such samples are awash in chemicals from their food, drugs and their metabolites, gut microbiota, environmental contaminants, and the like. Reliance on such comparisons is overly simplistic and can prove error prone, leading to erroneous and indeed even ludicrous “identifications” being put forward as facts in manuscripts submitted to journals.

Thus, despite efforts over many years toward better dissemination of the requirements for the exercise of critical judgment in the pursuit of metabolite identification via the publication of commentaries, perspective articles, and guidelines provided by, e.g., Metabolomics Society recommendations or indeed perspective articles from journal editors,^23−27^ poor metabolomics science is still published. Despite the original paper from the MSI^5^ being cited far in excess of 3000 times, clearly many groups do not follow this guidance rigorously, which we believe is due to a lack of understanding of the requirements for robust metabolite identification; as a result, submitted manuscripts and eventually a proportion of published papers are deeply flawed.

The situation is not helped by the fact that currently, and indeed for the foreseeable future, LC-MS is the major analytical technology in metabolomics, where the wide availability and ease of implementation of the technique has greatly reduced the barriers to entry into the field. Thanks to the efforts of the manufacturers, the instrumentation and associated metabolomics software tend to be very user friendly and make performing metabolic phenotyping relatively easy. Following data acquisition by LC-MS analysis and the production of a clean data matrix, after what is called data deconvolution, the next steps in the metabolomics experimental workflow are the data curation, data mining, and biochemical interpretation, which are significantly more demanding. These steps were once the tasks of knowledgeable experts; however, the availability of bespoke software and online tools that offer several utilities at the press of a key, or click of a mouse, has lowered the need to collaborate with such experts. Unfortunately, used uncritically as a “black box”, such tools allow access to the ever-expanding public libraries and databases that contain many thousands of spectra, identities, and some biochemical data. The truth is such that databases also contain many chemical entities not originating in the biosphere. For example, major databases that serve as the root source of many searches, e.g., the Human Metabolome Data Base (HMDB) or the NIST libraries, contain many synthetic industrial chemicals, pharmaceuticals, pesticides, food additives, etc. So, investigators who are not thorough and uncritically take the first “hit” may “identify” as “biomarkers” compounds that simply do not exist in the biochemistry of the specimen under analysis. More subtle errors can occur when, e.g., phytochemicals are “identified” as mammalian “biomarkers”. So, while the use of these resources is very tempting, if not performed critically, with an eye on “biological plausibility,” it may result in the authors committing major errors of data interpretation. Such inadequate annotation of “biomarkers” and pathways then leads to the construction of erroneous hypotheses based on them.

Before offering some examples of easily identifiable errors in metabolite identification, we should remind readers of some factors that should be common knowledge for those active in bioanalysis and MS. It is well established that LC-MS data collected on one instrument, operating using instrument settings optimized for that application, can vary significantly from data collected on different mass spectrometers operating under different ionization conditions. Such differences can be exacerbated by the use of different mobile phase compositions. Also, ion generation in ESI is not always as reproducible as one would like with factors such as, e.g., ion suppression/enhancement and varying adduct chemistry also coming into play. In fact, we have previously demonstrated that two mass spectrometers (QTOF-MS and QTRAP technologies) simultaneously analyzing the same LC effluent will reveal different metabolomic profiles highlighting different biomarkers.^28^ In addition, MS/MS mechanisms differ between instruments and analytical conditions. As a result, the analytical community cannot yet be confident that libraries produced in different laboratories and with different LC-MS instruments can be used elsewhere to establish trustworthy identification comparable to, e.g., those provided for GC-MS data which uses a far more predictable fragmentation pattern of an analyte due to the employment of electron ionization (EI).

Examples of erroneous identifications that contributed to manuscripts being rejected for publication as well as examples of similar errors found in published papers are provided in Tables 1 and 2 and are discussed below. They are, unfortunately, by no means unique and provided the motivation that prompted us to write this article. Most of the errors that we have categorized fall into one or other of two key criteria.

**Table 1:** Examples of Biologically Implausible Identifications Made Using LC/MS Platforms

Specimen analyzed	Analytes “identified” and their real uses
Biological fluid from an animal model (rat) of colon cancer	Bortezomib: Anticancer drug
	Adapalene: drug for treatment of acne
	Netilmicin: semisynthetic aminoglycoside
	Ibutilide: antiarrhythmic agent
	Varenicline: used to help people stop smoking
	Flecainide: used to prevent and treat abnormally fast heart rates
	
Treated cell culture	Disodium phosphate: salt that may be present in the sample but is not detected in RPLC/MS
	Pyrophosphate: an ion that maybe present in the sample but is not detected in RPLC/MS
	Dapsone hydroxylamine: a derivative of an anti-inflammatory and antibacterial drug
	Lansoprazole: a synthetic drug used to reduce gastric acid concentrations
	Imazamethabenz: a pesticide
	
HepaRG cells	Eprosartan: an angiotensin II receptor antagonist used for treatment of high blood pressure
	Forskolin: an antiglaucoma drug
	Phytochemical natural products: stilvenoids, aminoglycosides, and other natural products characteristic of diverse and different plants and fruits such as Valeriana, Blue Spur flowers, chicory, avocado, Chinese herb cortex Lycii, and terpenes found in various herbs and spices
	
Cell culture	Carbaryl: an insecticide
	Naproxen: a nonsteroidal anti-inflammatory drug
	O-Desmethylnaproxen: a naproxen metabolite
	Ramipril: an ACE inhibitor
	Dextromethorphan: an antitussive drug
	Lisinopril: an ACE inhibitor
	Primaquine: a medication to treat or prevent malaria
	Molsidomine: a withdrawn cardiovascular drug
	Tamoxifen: a selective estrogen receptor modulator synthetic

**Table 2:** Examples^b^ of Identifications Showing Unrealistic Elution Orders in RPLC Systems^a^

	*t*_R_ (min)^c^	Metabolite names	Characteristic log *K*_ow_ values^d^
Example 1	6.83	*Palmitic acid*	6.96
	8.11	Aspartic acid	–4.32
	8.29	*LysoPC* (15:0)	
	9.53	Lactic acid	–0.65
			
Example 2	1.68	*Linoleic acid*	7.51
	3.33	Citric acid	–1.67
	3.84	Uric acid	–1.46
	4.02	*Corticosterone*	1.99
	4.54	1-Methyladenosine	
	4.78	Galactonic acid	–1.87
	4.82	Glutamine	
	5.07	*SM(d*18:1/22:0)	
	5.55	Glutamate	–3.83
	6.45	*Chenodeoxycholic acid*	5.06
	6.69	Pyruvic acid	–1.24
	6.83	*Palmitic acid*	6.96
	7.42	*Arachidonic acid*	8.07
	7.86	*LysoPC* (17:0)	
	8.11	Aspartic acid	–4.32
	8.29	*LysoPC* (15:0)	
	9.33	Lactic acid	–0.65

a Apolar analytes are written in italics, more polar analytes in normal font.

b Studies are anonymized.

c The numbers are reproduced as found in their sources, but in some cases, *t*_R_ values have been reduced to two digits.

d Log *K*_ow_ were obtained from Chemspider. RPLC theory dictates that an increase in analyte log *K*_ow_ results in an increase in *t*_R_. Here, this relationship is not observed, and values are clearly in disarray.

### Low Biological Plausibility

This error is the result of identifying features as molecules that are completely “alien” to the specimens being analyzed, with their apparent presence in the samples being unprecedented or not explained. For example, we have come across many examples of the identification, sometimes as potential biomarkers of disease, of obscure phytochemicals, pesticides, or pharmaceuticals in human cell culture or synthetic chemicals in plant tissue culture. These “xenobiotics” which are “identified” as potential biomarkers should cause alarm bells to ring in the minds of researchers and should not be accepted without supporting evidence, comment, and explanation. Such errors are potentially characteristic of “sloppiness” in data curation, interpretation, and article preparation. It is the duty of authors to check for the plausibility of their “findings”. A database “hit” is not a confirmed identification but merely an indication of a possibility and does not, in our opinion, even constitute “annotation” let alone identification. Characteristic examples of such mistakes are shown in Table 1.

As indicated in Table 1, in one study, the analysis of a cell culture apparently identified 12 synthetic drugs, from a range of therapeutic areas. Similarly, another study using RPLC-Orbitrap-MS for the analysis of HepaRG cells reported as “biomarkers” two drugs and a number of natural products, including terpenes characteristic of diverse plants, spices, etc. It is not only cell culture studies that are redolent with xenobiotics, as shown in the example from an animal rat model of colon cancer where six synthetic pharmaceuticals, of varying action (anticancer, heart protection, acne treatment), were detected in biofluids. Along with these illogical IDs, two types of phosphate ions were detected in one of the studies shown in Table 1, which would not be detected in scanning mode LC-MS analysis. Unfortunately, these errors are not atypical, and the literature is regrettably replete with such examples.

### Unrealistic Chromatographic Properties

In many cases only the mass spectral data are compared against databases, and the LC data are disregarded. This is unfortunate as these putative annotations/identifications fail to comply with basic chromatographic rules and physicochemical properties. In most cases, this is evidenced with analytes reported in unrealistic elution order; in other cases, papers report enantiomer separations despite using nonchiral systems. Examples of nonlogical elution include (1) thymine eluting after aldosterone in RPLC, (2) an analyte and its glucuronide eluting in the same *t*_R_ (presumably reflecting in source fragmentation of the glucuronide), (3) iodide, phosphoric acid, and sulfuric acid detected on RPLC-MS, as well as the phosphate ions as reported in Table 1 (these examples have been seen in various papers), (4) palmitic acid with a *t*_R_ of less than 1 min when phosphocholine had a *t*_R_ > 10 min, (5) betaine at a *t*_R_ > 21 min despite eicosapentanoic acid eluting at *t*_R_ < 12 min in RPLC, and (6) LysoPC with a *t*_R_ = 10 min and triethylamine (a widely used LC eluent additive with negligible retention on RPLC) eluting in the same system at 22 min. Avoiding such profound mistakes necessitates a good understanding of basic principles and practice of liquid chromatography, as even a limited understanding of chromatographic principles would help the authors to identify obvious pitfalls/errors in identification, especially with regard to their elution order.

Characteristic examples of such mistakes for RPLC-MS analysis are provided in Table 2 and in the Supporting Information table, and similar examples could also be found for HILIC (hydrophobic interaction liquid chromatography). Thus, consideration of elementary factors controlling retention in RPLC such as the octanol–water partition coefficient (log *K*_ow_ or log P) and the need to suppress the ionization of acidic or basic groups provides a good guide to likely retention properties. For the examples shown in Table 2, characteristic log *K*_ow_ values are included which reflect molecular hydrophobicity and in this sense are directly related to the analyte retention time. As such, log *K*_ow_ values are relatively easy to calculate, using readily available software, and in fact, they are frequently used in retention prediction approaches. For RPLC, analytes with low log *K*_ow_ values are associated with short *t*_R_ (while they would indicate well retained analytes using HILIC) and high log *K*_ow_ values provide correspondingly longer *t*_R_ values (and the reverse is true for HILIC). Obviously, in the examples highlighted in Table 2, this correlation of log *K*_ow_ with elution is not seen. Thus, in these examples, analytes are shown in an obviously illogical order, with polar and apolar (nonpolar) analytes intermingled, and the elution order is improbable (e.g., aspartic and lactic acid eluting after palmitic acid in example 1).

The most egregious error for all of these studies, however, is the failure of the authors to, wherever possible, obtain authentic standards of compounds in order to confirm identities on which they later base a hypothesis, especially when these standards are often readily available at modest cost.

### Proposed Steps

Some basic housekeeping could radically improve the situation and some simple ideas are listed below.

1. Managers of databases for metabolomics should indicate when a compound in the database is entirely synthetic (industrial chemical, biocide, pharmaceutical etc.) and not known to be produced by any living organisms (e.g., mammals, plants, microbes etc.) or at least unlikely to be present due to ingestion of said compound. Where a compound has been shown to exist in nature, it could be explicitly linked to the class of organisms that produce it (e.g., microbes, plants, fungi, mammals etc.), and it should be made clear if it has never been confirmed as being present in other phyla.
  The investigator could be provided with the option to control the output by specifying, e.g., only mammalian/human in the origin in the search results. For example, the HMDB states for certain metabolites this: Metabolite X is not a naturally occurring metabolite and is only found in those individuals exposed to this compound or its derivatives. Technically X is part of the human exposome. The exposome can be defined as...; this explanation is a helpful policy that could provide an explicit warning to the investigator, but unfortunately is not provided for all DB entries.
2. Instrument manufacturers need to support and implement similar improvements to their own databases and those sold through their companies such that a consistent set of information on metabolite origin, biological or otherwise, is available to all researchers. A united effort to improve metabolite identification and make it easy or even automatic to characterize the quality of the metabolite identification and allow users to filter out obviously erroneous options will have a significant effect on the field.
3. Implement additional data into databases such as log(*P*) or *t*_R_ values to allow users and even software to help identify problematic metabolite identifications. While it may be challenging to come up with an accurate value of *t*_R_ for metabolites given the various chromatographic conditions used across the metabolomics field, some simple approaches could be used; after all, for the same combination of mobile phase and stationary phase, the elution order is generally the same. For example, reporting the *t*_R_ for samples run under typical RP conditions would go a long way toward identifying the very obvious errors seen in the examples provided in Table 2 and in the Supporting Information. Reference values could be generated and even software developed to translate standard *t*_R_ values to usable ranges for other types of chromatic separation conditions. Note that in GC-MS *n*-alkanes are spiked into samples to define a retention index scale and thus compensate for any t_R_ drift.^29^
4. Authors must embrace, and use, the experimental methods and guidelines for metabolite identification, such as those mentioned above, reporting the confidence level to which metabolites discussed in their papers have been identified (e.g., MSI Levels 1–4) with supporting evidence, which could be placed electronically in the Supporting Information. Authors must take all possible measures to increase confidence in the proposed identification before going forward to biochemical pathway analysis and hypothesis building. Otherwise, there is a major risk of wasting time and resources.
5. Reviewers need to carefully examine the metabolite identifications, especially those used to support any hypotheses developed in the manuscript and ensure that the information supplied is convincing and supported by detailed information (Supporting Information requires particularly careful examination). Any potential/actual problems in the area of metabolite identification should be highlighted to the editor of the journal.
6. Editors are responsible, ultimately, for the decision to publish. In a multidisciplinary area such as metabolomics, the reviewers (and even the editors themselves) selected may be experts in the topic under study (e.g., a disease state), but not metabolomics, and may fail to identify problems with metabolite identifications. Editors should aim to ensure that at least one of the reviewers has experience in the analytical aspects of metabolic phenotyping and is given the specific remit of ensuring that results published in the journal are at least plausible to someone “knowledgeable in the art”.
7. Journals/Publishers must have policies in place that explicitly encourage best practice in publishing metabolomic data, particularly with regard to metabolite identification and should provide clear guidance to authors in their instructions to authors about minimum requirements. This may result in additional documentation and data being necessary in manuscript handling. But, it will ultimately safeguard the status and reputation of the publisher’s end product.
8. Societies, regulators, and public bodies need to update and enforce guidelines in order to create an environment where metabolite identification in metabolic phenotyping studies is performed to agreed minimum standards. This effort will ensure that the data generated by grant-funded studies are of value. Action is also necessary to train policy makers, grant awarding bodies, and other interested parties to set these minimum standards. In this respect, recent activities such as those from mQACC and the Lipidomics community are most welcome.
9. Readers represent the final line of defense against poor metabolite identification. If, on reading a paper, a reader sees that it contains examples of poor work in this area the reader is advised to write to the journal and make concerns clear.

## Conclusion

Since the very first metabolic phenotyping publications in the 20th century which are recognized as having a metabolomics focus, much progress has been made in this field. However, one of the problems, possibly the largest, that limits acceptance of metabolic phenotyping investigations is a lack of confidence in the data. Such a situation does not exist to the same extent for genomic/transcriptomic or proteomic data where identification is easier (with perhaps the exception of protein post-translational modifications). We believe that improving the standard of metabolite identification and reporting is essential to remedy this situation which will go a long way toward confirming the inherent value of metabolomics.

## Supporting Information Available

Table S1: Examples of identifications showing unrealistic elution orders in RPLC-MS systems, containing data from four examples of poor identification (PDF)
