# Supplementary material for: Ensuring Fact-Based Metabolite Identification in Liquid Chromatography–Mass Spectrometry-Based Metabolomics
Source: Anal Chem. 2023 Feb 15;95(8):3909–16. doi: 10.1021/acs.analchem.2c05192 (PMC9979140; doi:10.1021/acs.analchem.2c05192)
Supplement: Supplementary file 1 — ac2c05192_si_001.pdf [file ac2c05192_si_001.pdf]

Supporting Information

Ensuring Fact-Based Metabolite Identification in LC-MS-Based Metabolomics

Georgios Theodoridis* [1,2,3], Helen Gika[2,3,4], Daniel Raftery[5,6], Royston Goodacre[7], Robert S. Plumb[8], Ian D. Wilson*[7,9]

[1] Department of Chemistry, Aristotle University of Thessaloniki, 54124, Greece

[2] Biomic AUTh, Center for Interdisciplinary Research and Innovation (CIRI-AUTH), Balkan Center B1.4, 10th km Thessaloniki-Thermi Rd, P.O. Box 8318, GR 57001 Thessaloniki, Greece

[3] FoodOmicsGR, AUTh node, Center for Interdisciplinary Research and Innovation (CIRI-AUTH), Balkan Center B1.4, 10th km Thessaloniki-Thermi Rd, P.O. Box 8318, GR 57001 Thessaloniki, Greece

[4] Laboratory of Forensic Medicine and Toxicology, Department of Medicine, Aristotle University, 54124 Thessaloniki, Greece.

[5] Northwest Metabolomics Research Center

[6] Mitochondria Metabolism Center, Anesthesiology and Pain Medicine, University of Washington, Seattle, WA 98109

[7] Centre for Metabolomics Research, Department of Biochemistry and Systems Biology, Institute of Systems, Molecular and Integrative Biology, University of Liverpool, BioSciences Building, Crown St., Liverpool, L69 7ZB, UK

[8] Scientific Operations, Waters Corporation, IMMERSE, Cambridge, 02142, MA, United States

[9] Division of Systems Medicine, Department of Metabolism, Digestion and Reproduction, Imperial College London, Hammersmith Campus, London, W12 0NN, UK

* correspondence gtheodor@chem.auth.gr,
i.wilson@imperial.ac.uk

Table S1: Examples# of identifications showing unrealistic elution orders in RPLC-MS systems*

	RT(min)	Metabolites Names and characteristic LogKow values
Example 1	2.99	*LysoPC(15:0)*
	3.41	Phenylalanine
	3.62	Glucose
	4.27	Citric acid
	9.14	Lactic acid
	8.11	Aspartic acid -4.32
	8.29	*LysoPC(15:0)*
	9.53	Lactic acid -0.65
Example 2	4.27	*Palmitic acid* 6.96
	4.50	1-Methylhistamine
	4.60	5-Hydroxy-L-tryptophan
	4.86	L-Lysine -2.99
	5.08	*2-Hydroxydecanedioic acid*
	5.09	Asymmetric dimethylarginine
	5.09	4-Hydroxynonenal
	5.09	L-alpha-Aminobutyric acid
Example 3	2.09	Isocitric acid -2.01
	2.21	*Arachidonic acid 8.07*
	2.49	*18-Hydroxycorticosterone*
	3.55	Glucose -2.89
	3.67	*11-Dehydrocorticosterone 1.77*
	4.45	L-Leucine
	5.09	D-Ornithine
	5.24	Mevalonic acid-5P
	5.31	Glycine -3.41
	6.91	*Eicosadienoic acid 8.50*
	7.56	Deoxyuridine -1.19
Example 4	3.37	*PC(18:1(9Z)/0:0)*
	3.76	*Arachidonic acid 8.07*
	5.21	*Lysopc(16:1(9Z)/0:0)*
	11.79	*Lysopc(18:1(11Z))*
	13.18	L-Asparagine -4.99
	13.22	*PC(18:2(9Z,12Z)/18:1(11Z))*
	17.95	Glutathione
	21.13	Guanosine diphosphate -5.55

*Apolar analytes = italics, more polar analytes = normal font

LogKow were obtained from Chemspider. RPLC theory dictates that increase in analyte log Kow results to increase in t_R. Here this relationship is not observed and values are clearly in disarray.

The numbers are reproduced as found in their sources but in some cases t_R values have been reduced to 2 digits.
[#]Studies are anonymised.
